# Predatory and competitive interaction in *Anopheles gambiae* sensu lato larval breeding habitats in selected villages of central Uganda

**DOI:** 10.1186/s13071-021-04926-9

**Published:** 2021-08-21

**Authors:** Hudson Onen, Robinson Odong, Moses Chemurot, Frédéric Tripet, Jonathan K. Kayondo

**Affiliations:** 1 Department of Zoology, Entomology and Fisheries Sciences, College of Natural Sciences, School of Biosciences, Makerere University, PO Box 7062, Kampala, Uganda; 2 Department of Entomology, Uganda Virus Research Institute (UVRI), PO Box 49, Entebbe, Uganda; 3 Centre for Applied Entomology and Parasitology, School of Life Sciences, Keele University, Keele, SFD ST5 5BG UK

**Keywords:** Aquatic insects, *An. gambiae* s.l. competition, Habitat types, Macroinvertebrates, Predation, Niche overlap

## Abstract

**Background:**

Malaria is often persistent in communities surrounded by mosquito breeding habitats. *Anopheles gambiae* sensu lato exploit a variety of aquatic habitats, but the biotic determinants of its preferences are poorly understood. This study aimed to identify and quantify macroinvertebrates in different habitat types with determined water physico-chemical parameters to establish those preferred by *An. gambiae* s.l. larvae as well as their predators and competitors.

**Methods:**

A field survey was conducted in Kibuye and Kayonjo villages located in the vicinity of the River Sezibwa, north-eastern Uganda to identify Anopheline larval habitats shared by aquatic insects. Habitats were geo-recorded and as streams, ponds, temporary pools and roadside ditches. From October to December 2017, random microhabitats/quadrats were selected from each habitat type, their water physico-chemical parameters (electrical conductivity, total dissolved solids, temperature and pH) were measured, and they were sampled for macroinvertebrates using standard dippers. All collected arthropod macroinvertebrates were then morphologically identified to family level and enumerated.

**Results:**

Principal component analysis showed that the four larval habitat types were characterized by distinct physico-chemical parameter profiles. Ponds and streams had the highest number and diversity of macroinvertebrate insect taxa and sustained few *An. gambiae* s.l. larvae. *Anopheles gambiae* s.l. were more common in roadside ditches and particularly abundant in temporary pools which it commonly shared with Dytiscidae (predaceous diving beetles) and *Culex* spp. Cluster correlation analysis conducted on the abundance of these taxa within quadrats suggested that *An. gambiae* s.l. and Dytiscidae have the most similar patterns of microhabitat use, followed by Cybaeidae (water spiders). Whilst *Culex* spp. co-occurred with *An. gambiae* s.l. in some habitats, there was only partial niche overlap and no clear evidence of competition between the two mosquito taxa.

**Conclusions:**

Ponds and streams are habitats that host the largest diversity and abundance of aquatic insect taxa. *Anopheles gambiae* s.l. larvae distinctively preferred temporary pools and roadside ditches, where they were exposed to few predators and no apparent competition by *Culex* spp. Further studies should aim to test the impact of Dytiscidae and Cybaeidae on *An. gambiae* s.l. dynamics experimentally.

**Graphical Abstract:**

**Figure:**
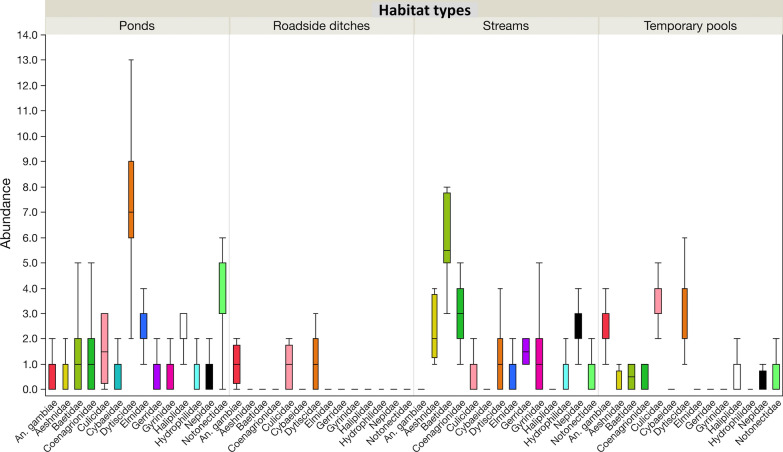

**Supplementary Information:**

The online version contains supplementary material available at 10.1186/s13071-021-04926-9.

## Background

Sub-Saharan African countries account for approximately 90% of the global malaria cases and deaths [1]. Factors responsible for the persistence and prevalence of malaria in sub-Saharan Africa include the availability of human hosts and favourable conditions for mosquito vector populations, including high temperature, humidity, rainfall and an abundance of aquatic breeding habitats [2, 3]. Since the implementation of Roll Back Malaria, the widespread use of insecticide-treated nets (ITNs) and indoor residual spraying (IRS) has played a significant role in reducing mortality and morbidity in malaria-endemic regions [4]. However, the intervention’s effectiveness is threatened by the fast spread of resistance to most chemical insecticides available for public health, change in vector species composition and a shift in vector biting behaviour [5]. Outdoor biting mosquitoes are responsible for residual malaria transmission in many parts of sub-Saharan Africa and pose new challenges, as they are not sufficiently controlled using conventional tools [6].

Therefore, there is an urgent need to improve larval source management (LSM) by considering aquatic insect predators as a scalable tool for integrated vector management (IVM) programs to reduce malaria vector populations [7–9]. To be successful, IVM programs using LSM would benefit from in-depth knowledge of the mosquito larval ecology and a better understanding of the trophic interactions in larval habitats. This could prioritize larval control efforts focusing on habitats that sustain the highest densities of anopheline larvae while protecting habitats rich in natural predators. A better understanding of prey–predator spatio-temporal dynamics could also lead to discovering and adding new biocontrol tools in the fight against malaria. Finally, assessing larval habitat preferences, the extent of niche overlap and possible competitive interactions between mosquito taxa is also relevant to predicting possible changes in mosquito communities and disease transmission in response to vector control interventions. It can also help understand the possible consequences of suppressing the malaria mosquito, *Anopheles gambiae* sensu lato, on aquatic predators and competitors in ecosystems [10].

Across many regions of Africa, mosquito species within the *An. gambiae* s.l. complex are responsible for the majority of malaria transmission [11]. The species in the complex are *An. amharicus*, *An. arabiensis*, *An. bwambae*, *An. coluzzii*, *An. gambiae* sensu stricto, *An. melas*, *An. merus*, *An. quadriannulatus* and *An. fontenillei* [12–14]*.* Their respective spatio-temporal distribution and ecological niches are largely determined by patterns of temperatures, rainfall and physico-chemical parameters of their preferred breeding sites [2, 15]. For instance, *An. bwambae* breeds in brackish geothermal spring water [16], while *An. melas* and *An. merus* are often associated with saline waters [17]. Among the freshwater-adapted species, *An. gambiae* s.s. and *An. arabiensis* prefer temporary sunlit habitats such as ground puddles, tire tracks and hoof prints, while *An. coluzzii* are mainly found in more permanent and sometimes larger artificial water bodies such as rice fields [18, 19].

The different breeding habitats of the *An. gambiae* s.l. species complex varies in the predator and competitor taxa they support [20]. Compared to temporary habitats, permanent breeding habitats tend to support higher densities of predator and competitor taxa [21]. Predation on larvae by aquatic insect predators affects larval development, impacts the adult sex ratio at emergence and subsequently influences the adult life-history traits such as body size, fecundity and longevity [22, 23]. The presence of predators and competitors in aquatic larval habitats lowers their chances of being chosen as oviposition sites by female *An. gambiae* s.s. [24, 25]. Such adaptive responses would, therefore, ensure that females lay eggs in habitats that maximize their offspring's survival [26].

*Anopheles coluzzii* is restricted to West and Central Africa; hence, in East African countries such as Kenya [27, 28], Uganda [29] and Tanzania [30, 31], *An. gambiae* s.s. and *An. arabiensis* are the dominant species of the *An. gambiae* species complex and are present in various lowland and highland breeding habitats [32]. On the other hand, *An. merus* is found in Kenya and Tanzania's coastal areas [33, 34]. As in West Africa, the larval breeding habitats of *An. gambiae* s.s. and *An. arabiensis* are also inhabited by various aquatic insect predators and larvae from other mosquito genera such as *Culex* and *Aedes* [35]. Larvae are sometimes also found together with those from the *An. funestus* complex, although the latter tend to prefer small spring-fed pools, medium-sized natural ponds and slow-moving waters along river tributaries [36]. Depending on breeding habitats and their physico-chemical parameters, other less common mosquito species can be found together with *An. gambiae* s.l. For example, in Northern Tanzania, other anophelines and Aedes spp. are sometimes found [20].

In north-eastern Uganda and the Lake Victoria Basin (LVB) region, some villages have a high abundance of *An. gambiae* s.s. in various commonly occurring breeding habitats, including streams and standing waters [37, 38]. Although some regions in East Africa have similar ecologies, the communities of aquatic predators and competitors of *An. gambiae* s.l. are poorly known. This study attempted to fill this gap by determining the diversity and abundance of potential predators and larval competitors of *An. gambiae* s.l. as well as their associations in ponds, roadside ditches, temporary pools and streams with the determined water physico-chemical parameters in Kibuye and Kayonjo villages located near River Sezibwa in north-eastern Uganda. These findings were important for assessing the relevance of potential predatory and competitive interactions between aquatic macroinvertebrates and larvae of the malaria vectors. This information could inform vector control strategies targeting larval habitats through physical or biocontrol approaches and lead to novel biocontrol approaches to complement current malaria vector control programs.

## Methods

### Study area

**Fig. 1:**
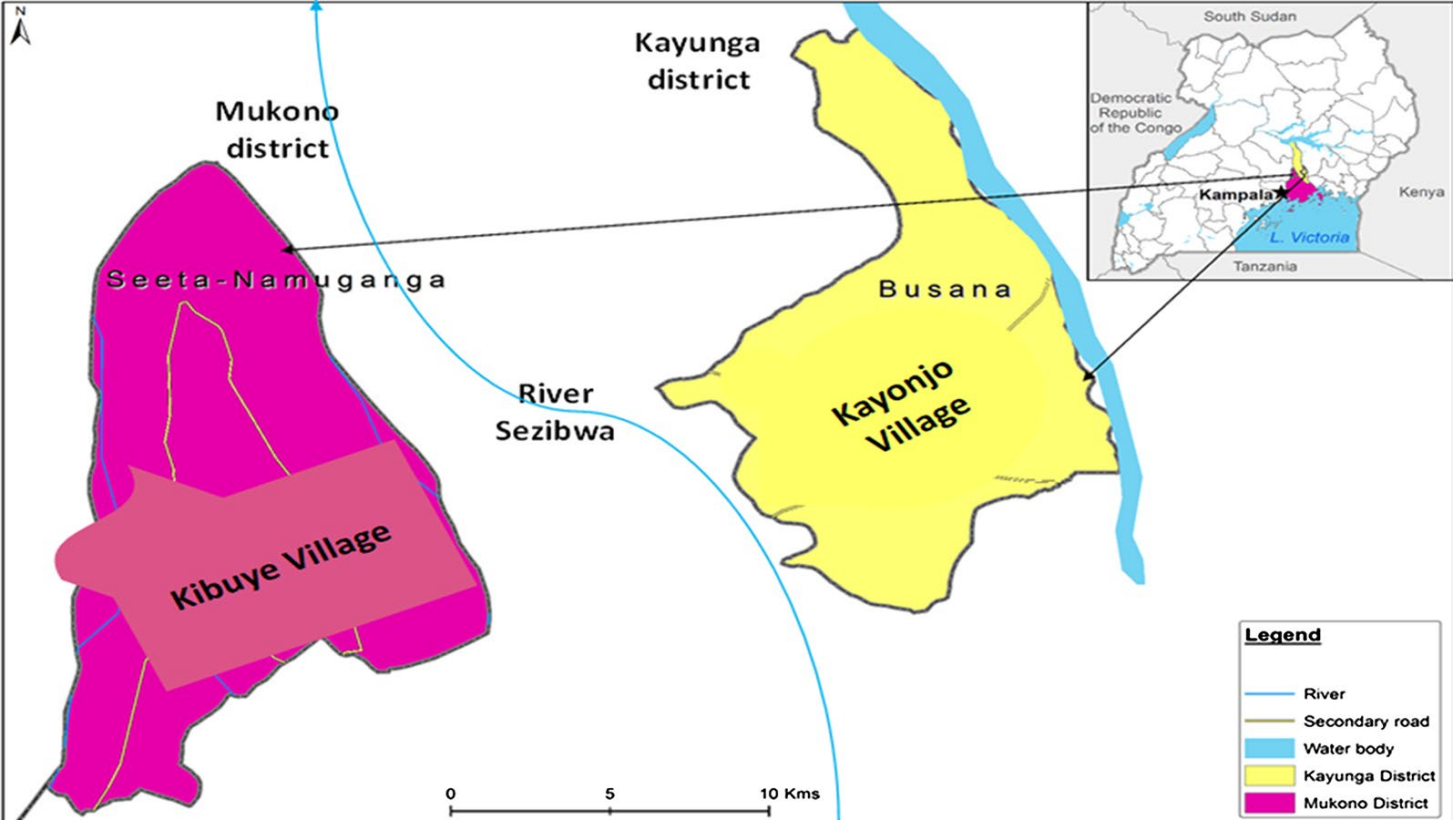
Location of the study sites in Mukono and Kayunga districts, Uganda. Source: Google Earth and field survey coordinates. The map was created using QGIS and publicly available shapefiles from QGIS website (https://qgis.org/en/site/)

This study was conducted in Kibuye and Kayonjo villages situated along the River Sezibwa of central Uganda from October to December 2017. Kibuye is located on the western side of the riverbank in the Mukono district, while Kayonjo is on the Kayunga district's eastern bank (Fig. 1). Kibuye and Kayonjo are approximately 33 km apart, 68 and 81 km from Kampala (Uganda's capital city), respectively. Mukono lies within 0°21′17.99″ N, 32°45′07.57” E, with a total human population of 596,804 on 1875.1 km^2^ of land cover [39]. With a total human population of 294,613 on 1810 km^2^ of land cover, Kayunga lies within 0°45′59.29″ N, 32°59′00.47″ E. Both districts are predominantly covered by savannah vegetation [40] and experience two rainy seasons in a year (wet seasons: March–May, September–December; dry seasons: June–October, December–February). The average annual temperature in Kayunga and Mukono district is 21.5 °C [41]. The main economic activities in the study area are fishing and subsistence agriculture.

### Characterization of mosquito breeding habitats

**Fig. 2:**
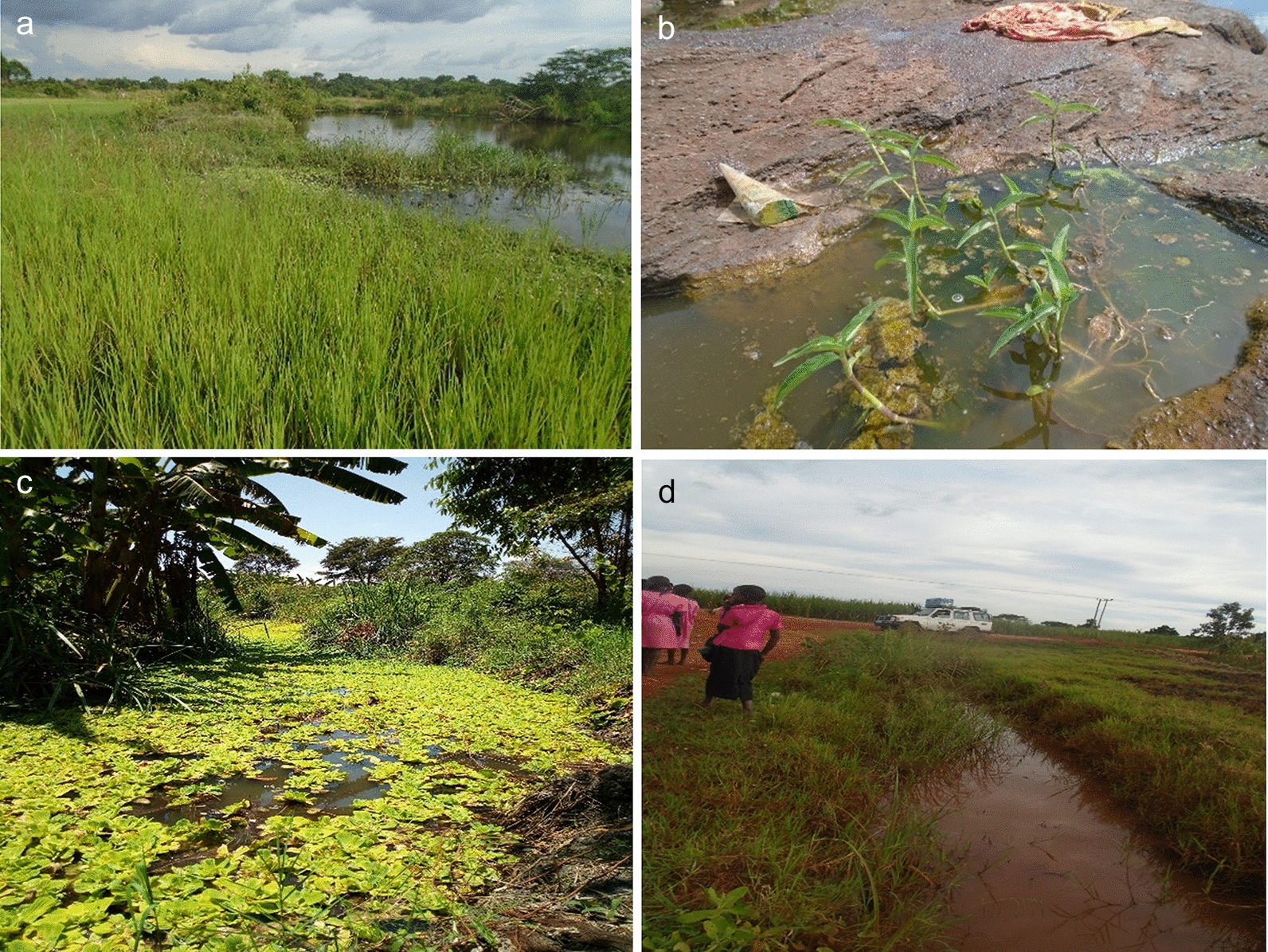
Stream, temporary pool, pond and roadside ditch mosquito and macroinvertebrate larval habitats (**a**, **b**, **c** and **d**). Larval habitats where samples were taken from randomly selected quadrats after determination of water physico-chemical parameters

Field surveys were conducted in Kibuye and Kayonjo villages in July 2017 to identify the potential breeding habitats of *An. gambiae* s.l. and associated macroinvertebrates [35]. Geographical coordinates of habitats were recorded using a Global Positioning System (Shenzhen Pengjin Technology Co., Ltd., Shenzhen, China) receiver, and their average surface areas (m^2^) were estimated. Based on the survey results, four habitat types were identified in each village and categorized as follows: streams (i.e., naturally slow-running water bodies with diversions of stagnant water from the mainstream which may not dry off in the absence of rainfall); ponds (non-flowing water collected in artificial pools that may not dry off within 6 months in the absence of rainfall); temporary pools (pools of water collected often within rocks and other brick pits which could persist for at least a month in the absence of rainfall); and roadside ditches (pools of water often collected along the road pavement that could persist for at least 2 weeks in the absence of rainfall) (Fig. 2).

### Determination of water physico-chemical parameters

From October to December 2017 and for each category of habitat type, four larval habitats in each village were sampled monthly over 4 days of fieldwork (32 independent habitats surveyed). A total of eight 1 × 1 m quadrats were randomly selected from each habitat type, totaling 256 quadrants. Therefore, a total of 768 quadrats were surveyed over the 3-month study.

For each quadrat sampled, the electrical conductivity (EC) (μS/cm), total dissolved solids (TDS) (mg/l), pH and temperature (°C) were measured by dipping a calibrated “three-in-one” Netutal pocket pen-type TDS meter probe (Shanghai Hello Pure Water Treatment Technology Co., Ltd., Shanghai, China) and ATC [automatic temperature compensation] digital pH meter (Shanghai Hello Pure Water Treatment Technology Co., Ltd., Shanghai, China) in water. Measurements were conducted in situ in the morning (10:00–11:00) and afternoon (13:00–14:00), and averaged readings were recorded.

### Sampling and identification of predator and competitor macroinvertebrates

Each randomly picked quadrat for *An. gambiae* s.l. larvae and other aquatic insects was sampled by dipping standard 350 ml dippers (BioQuip Products, Inc., CA, USA) four times per quadrat. Samples collected from each quadrat in the morning and afternoon were combined and sieved using a stainless-steel mesh strainer (Innovative Lab Instruments, New Delhi, India). Sorting of invertebrates was done in the field, and samples were preserved in 5 ml falcon tubes (Fisher Scientific Ltd., UK) containing 80% ethanol. They were transported to the Entomology laboratory at the Uganda Virus Research Institute (UVRI) in Entebbe and identified by morphological characteristics to family level with the aid of a microscope (Opto-Edu Co., Ltd., Beijing, China) at ×40 eyepiece magnification. The identification of macroinvertebrates was carried out with the use of guides by Gerber and Gabriel [42] and Gill [43], while guides by Hopkins [44] and Rozeboom and Stone [45] were used for the identification of mosquito larvae. The identified individuals were counted.

### Data analyses

#### Criteria used for identification of the statistical unit

Particular attention was paid to identifying the unit of analysis appropriate for revealing possible interactions between aquatic insect taxa at the quadrats' microhabitat scale. Water physico-chemical parameters did not vary much between quadrats within a breeding site. This was verified using neighbour-joining clustering analysis based on all the four water physico-chemical parameters (TDS, pH, EC and temperature), which showed that randomly sampled quadrats from the same habitat tended to cluster together (Additional file 1: Figure S1a). Therefore, the mean from all quadrats from each habitat (*n* = 32) was used for all analyses to avoid pseudoreplication.

In contrast, data on aquatic insect taxa presence or absence, abundance and frequency (percentage of quadrats where the taxa were detected) were analysed using each quadrat as a statistical unit (*n* = 768). This was done under the assumption that stream, pond, temporary pool and roadside ditch larval habitats are spatially heterogeneous. Therefore, predatory or competitive interactions between aquatic insect taxa are best examined at the micro spatial scale of the quadrats—i.e., microhabitats within larval habitats. Neighbour-joining clustering based on all aquatic insect taxa's abundance demonstrated that randomly sampled quadrats taken from the same habitat did not cluster together, thereby highlighting the importance of variation within habitats (Additional file 1: Figure S1b).

#### Statistical analyses

All data obtained were analysed using JMP version 14 software (SAS Institute, Inc., USA). Data were checked for deviations from normality and heterogeneity, and analyses were conducted using parametric and non-parametric methods as appropriate. The Shannon–Weaver Index [46, 48] was used to measure macroinvertebrate diversity across villages, months of collections and habitat types. Multivariate analysis using principal component analysis (PCA) was conducted to highlight differences in water physico-chemical parameter profiles (pH, TDS, EC and temperature) across habitat types. Spearman's rank correlation test was conducted to examine possible associations between the abundance of *An. gambiae* s.l. and that of macroinvertebrates across and within the four habitat types. Cluster correlation analysis was used to highlight further similarities in each taxa profile of correlation in abundance with other species, hence overlapping habitat and microhabitat use. Finally, the cluster membership data generated for each taxon and the three habitats most commonly used by *An. gambiae* s.l. was used for two-way higher-level hierarchical clustering analyses using neighbour-joining (Ward clustering).

## Results

### Physico-chemical parameters of aquatic habitat types

Overall, there was no significant difference in mean temperature, pH, TDS, or EC measured from each larval breeding site in relation to village (Kruskal–Wallis H-test: *χ*^2^ < 0.12, *df* = 1, *P* > 0.736 in all cases) and month of collections (Kruskal–Wallis H-test: *χ*^2^ < 2.73, *df* = 3, *P* > 0.257 in all cases). Predictably, clustering analysis showed that measurements taken at different time points or different quadrats from the same breeding site often clustered together (Additional file 1: Figure S1a). Consequently, subsequent analyses were conducted on the mean water physico-chemical parameters values per breeding site across all samples measured over the three months.

Streams, ponds, temporary pools and roadside ditches differed significantly in some of their water physico-chemical parameters (temperature, pH, TDS and EC) (Kruskal–Wallis H-test: *χ*^2^ > 25.2, *df* = 3*, P* < 0.001 for all four parameters). Roadside ditches had a significantly higher temperature than temporary pools, ponds and streams (Dunn pairwise comparisons test: *Z* > 3.7, *P* < 0.013 in all cases). The same applied to other water physico-chemical parameters such as pH (Dunn pairwise comparisons test: *Z* > 3.4, *P* < 0.042 in all cases), EC (Dunn pairwise comparisons: *Z* > 3.2 and *P* < 0.052 in all cases) and TDS (Dunn pairwise comparisons test: *Z* > 3.9; *P* < 0.03 in all cases).

**Fig. 3:**
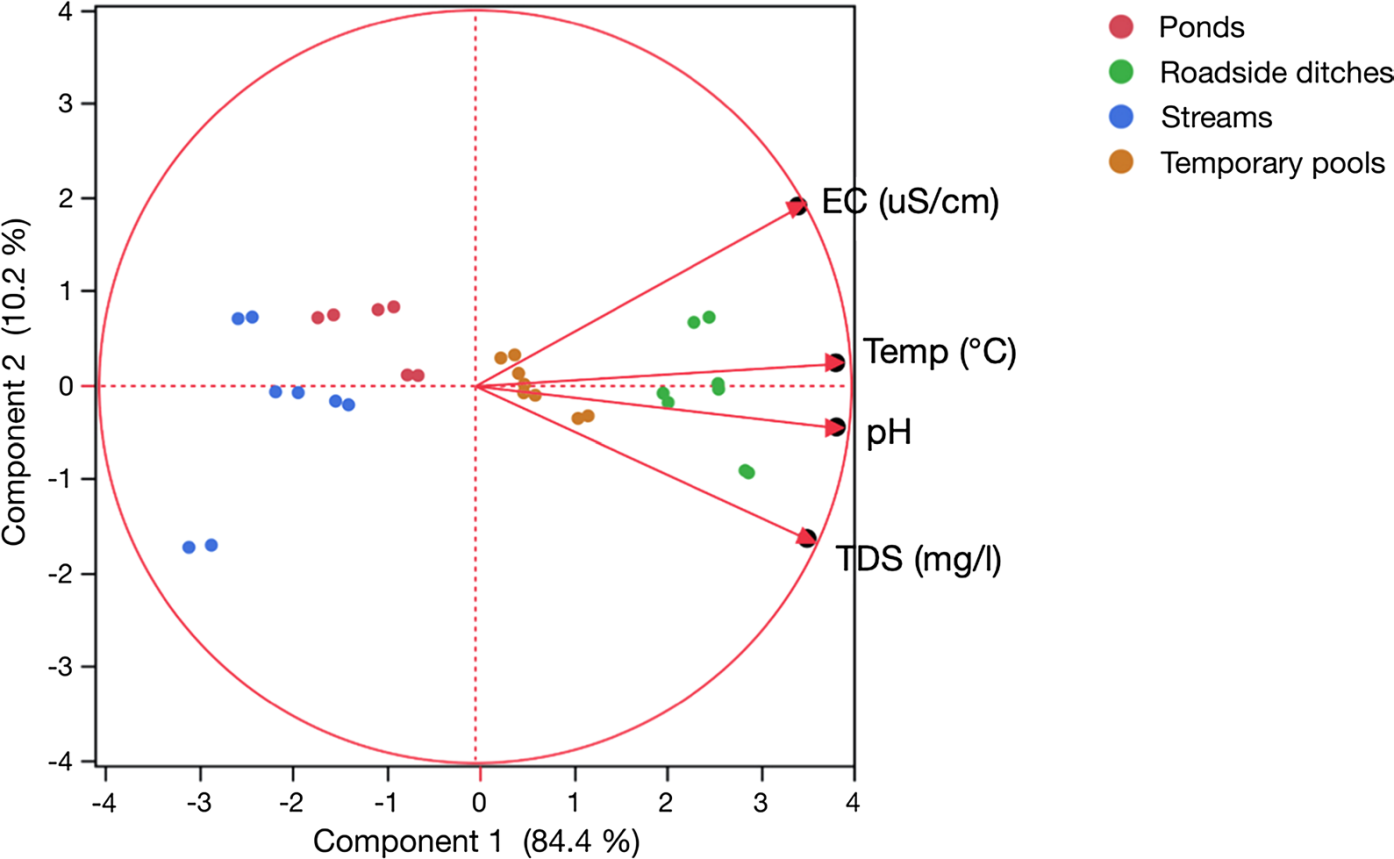
Principal component analysis (PCA) of water physico-chemical parameters across habitat types (stream, temporary pool, pond and roadside ditch). *EC* electrical conductivity, *TDS* total dissolved solids

The highest mean temperature was recorded in roadside ditches followed by temporary pools, ponds, and the lowest in streams. The same trends were also recorded with pH, EC and TDS. In habitats with lower mean temperatures, such as in streams and ponds, pH was slightly acidic, but in temporary pools and roadside ditches, which were generally warmer, pH was alkaline. Furthermore, EC and TDS values were low in streams and ponds but high in temporary pools and roadside ditches (Table 1). All variables were intercorrelated, with pH and temperature being the most intercorrelated (Spearman correlation test: *r*_(123)_ = 0.921, *P* < 0.001), while conductivity and TDS were the least (Spearman: *r*_(123)_ = 0.608, *P* < 0.001). PCA showed that physico-chemical parameters had high canonical and significant loadings on the first two components (Bartlett test on eigenvalues: *χ*^2^ > 23.0, *P* < 0.001 in both cases). All parameters contributed to component 1 (loading value range 0.861–0.963), which explained 84.4% of the variance. Additionally, conductivity and TDS had a medium positive (0.475) and negative (−0.406) loading on component 2 (10.2% of variance). The resulting PCA scores resulted in distinct clusters for the four aquatic habitats (Fig. 3).

**Table 1:** Mean and standard deviation of water temperature, conductivity, total dissolved solids and pH across habitat types

Habitat	Temperature (°C)	EC (uS/cm)	TDS (mg/l)	pH
Streams	26.3 ± 0.29	366.5 ± 35.36	470.9 ± 28.08	3.8 ± 0.38
Ponds	27.9 ± 0 .20	402.2 ± 6.05	478.4 ± 27.26	4.3 ± 0.39
Temporary pools	29.0 ± 0.15	415.3 ± 5.46	522.8 ± 15.44	6.4 ± 0 .72
Roadside ditches	30.8 ± 0.28	441.3 ± 13.03	556.6 ± 33.67	8.1 ± 0.13

*EC* electrical conductivity, *TDS* total dissolved solids

### Diversity of competitor and predator insect taxa in aquatic habitats

A total of 1428 macroinvertebrate specimens were collected from streams, ponds, temporary pools and roadside ditches in Kibuye and Kayonjo villages from October to December 2017. Of the collected specimens, 96 were anopheline mosquito larvae belonging to the *An. gambiae* s.l. complex, while 1332 were aquatic macroinvertebrates belonging to 14 families, including Culicinae mosquito larvae (Additional file 2: Table S1 and S2).

Overall, ponds and streams had the highest number of macroinvertebrate insect taxa, or taxa richness (*n* = 14), followed by temporary pools (*n* = 12) and roadside ditches (*n* = 11). Diversity (Shannon index) followed a similar pattern. The two villages had very similar profiles in terms of taxa present and diversity and did not differ significantly in median abundance of each taxon (Wilcoxon signed-rank test: *Z* = −223.00; *P* = 0.180). In addition, the median abundance of taxa for each habitat correlated strongly between the two villages (Spearman rank correlation test: ponds: *Rho* = 0.934*, P* = 0.001; streams: *Rho* = 0.992*, P* = 0.001; roadside ditches: *Rho* = 0.600*, P* = 0.023; and temporary pools: *Rho* = 0.945*, P* = 0.001). Further analyses were therefore performed across the combined data from both villages.

### Abundance and frequency of competitors and predator insect taxa and aquatic habitat type

Overall, the median abundance of macroinvertebrate insect taxa differed significantly between habitat types (Kruskal–Wallis H-test: *χ*^2^ = 28.0, *df* = 3,* P* < 0.001). Ponds and streams supported the highest numbers of aquatic insects (Additional file 2: Table S1 and S2) and did not differ significantly in median abundance (Dunn pairwise comparisons test: *Z* = 0.51, *P* = 1.00). Roadside ditches and temporary pools supported significantly lower aquatic insect abundance than the other two habitat types (Dunn pairwise comparisons test: *Z* > *2*.7, *P* < 0.032 in all cases).

**Fig. 4:**
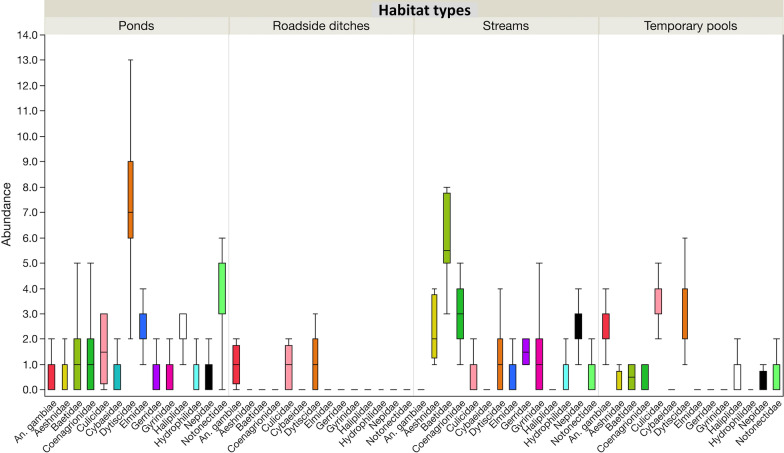
Variation in median abundance and quartiles of different aquatic insect taxa across habitat types (streams, temporary pools, ponds and roadside ditches)

Streams and ponds differed in their most abundant taxa and frequency. Few *An. gambiae* s.l. larvae were collected from ponds, and they were present in only 37.5% of pond quadrats in both villages. They were rare observations from quadrats from streams with less than 1% frequency in both villages. These habitats differed in the type of aquatic insect communities they supported. For example, ponds had the highest abundance of Dytiscidae which occurred at 100% frequency in both villages, followed by Notonectidae (backswimmers) which had an average frequency of 99.1% across both villages. In streams, Baetidae (minnow mayflies) recorded the highest abundance, followed by Coenagrionidae (narrow-winged damselflies) and Aeshnidae (darner dragonflies), with all the taxa occurring at 100% frequency in both villages. The preferred habitats for *An. gambiae* s.l. larvae were temporary pools and roadside ditches. They were detected in 99.1 and 75% of sampled quadrats in Kibuye and Kayonjo villages, respectively, and these habitats were shared with *Culex* spp. larvae at a frequency between 62.5 and 100%. Dytiscidae were the most abundant predators in these habitats, followed by Notonectidae, Haliplidae (crawling water beetles) and Elmidae (riffle beetles) (Additional file 2: Tables S1, S2 and Fig. 4).

### Temporal changes in the abundance of aquatic insect taxa

The abundance of some aquatic insect taxa in ponds, roadside ditches, streams and temporary pools varied significantly between months of collection (Kruskal–Wallis H-test: *χ*^2^ > 6.4, *df* = *2, P* < 0.05 in all cases). In ponds, Gyrinidae (whirligig beetles) and Hydrophilidae (water scavenger beetles) were significantly more abundant during the start and peak of the short rainy season in October and November (Dunn pairwise comparisons test: *Z* < −10.8*, P* < 0.001 in both cases). In contrast, Baetidae was significantly more common in December, the rainy season's end (Dunn pairwise comparisons test: *Z* = 2.5, *P* = 0.039). In roadside ditches, Aeshnidae, Baetidae, Coenagrionidae and Nepidae (water scorpions) increased in abundance towards the end of the rainy season (Dunn pairwise comparisons test:* Z* > 2.5, *P* < 0.039).

**Fig. 5:**
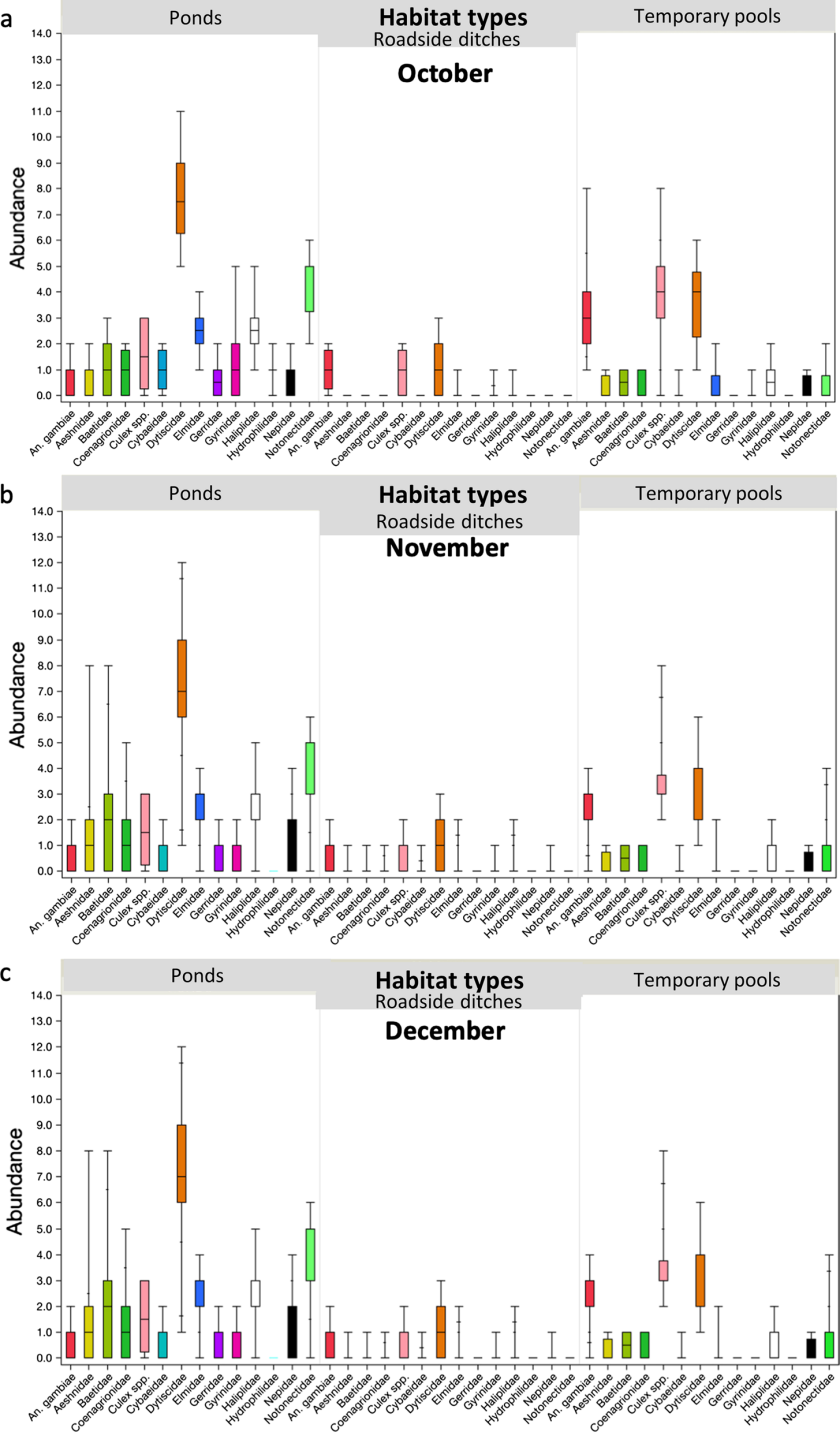
Temporal changes in the abundance of aquatic insect taxa in larval habitats (streams, temporary pools, ponds and roadside ditches) in October, November and December 2017

In streams, *An. gambiae* s.l. were more abundant in November at the peak of the short rainy season rather than at the start (Dunn pairwise comparisons test: *Z* < 3.9*, P* < 0.05), and the same was true for Hydrophilidae (Dunn pairwise comparisons test: *Z* < 10.9*, P* < 0.001). However, the collected number of Cybaeidae, Gyrinidae and Haliplidae significantly increased in December, the end of the short rainy season (Dunn pairwise comparisons test: *Z* > 3.0, *P* < 0.007 in all cases). In temporary pools, *An. gambiae* s.l. larvae, Culicidae larvae and Gyrinidae were significantly more abundant in October than in the following months (Dunn pairwise comparisons test*: Z* > −3.2, *P* < 0.004 in all cases) (Fig. 5).

### Variation in diversity in relation to habitat type and season

**Fig. 6:**
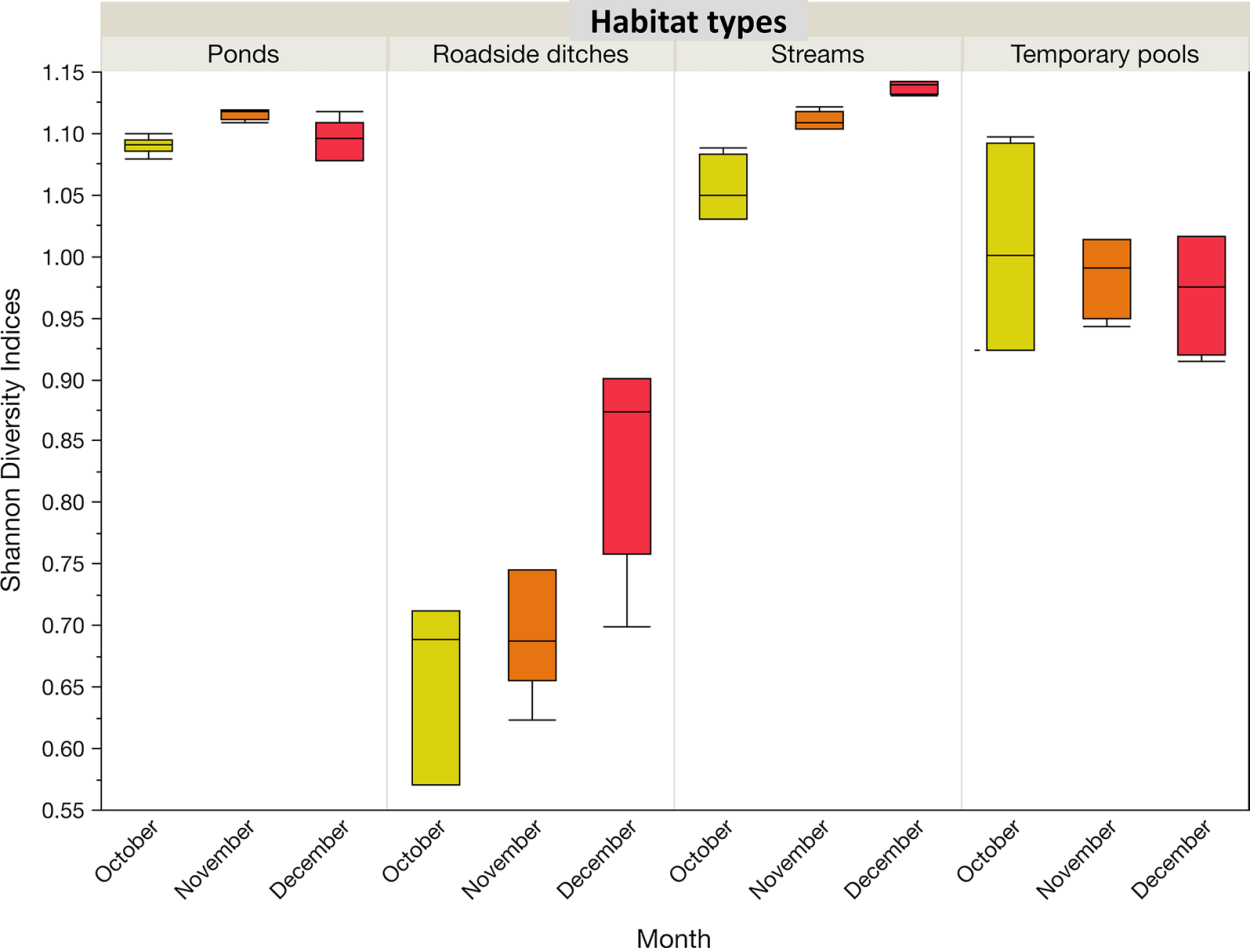
Variation in diversity indices (H′) of aquatic insect taxa across larval habitats (streams, temporary pools, ponds and roadside ditches) and months

The diversity of taxa (Shannon index) varied significantly across habitat types (Kruskal–Wallis H-test: *χ*^2^ = 429.3, *df* = 3*, P* < 0.001). On average, the highest diversity was recorded in streams (1.10, 1.10–1.12) followed by ponds (1.11, 1.07–1.14), temporary pools (0.99, 0.93–1.02) and roadside ditches (0.71, 0.65–0.76) (Additional file 2: Tables S1, S2 and Fig. 6). The diversity of insect taxa increased from November to December in ponds and roadside ditches (Dunn pairwise comparisons test: *Z* < 7.8, *P* < 0.001 in both cases), and from October to November in streams (Dunn pairwise comparisons test: *Z* = 6.5, *P* < 0.001), while in temporary pools, no significant increase was recorded (Wilcoxon rank-sum test: χ^2^ = 2.7*, df* = 2, *P* = 0.263) (Fig. 6).

### Association between *An. gambiae* s.l. larvae, predators and competitors

**Fig. 7:**
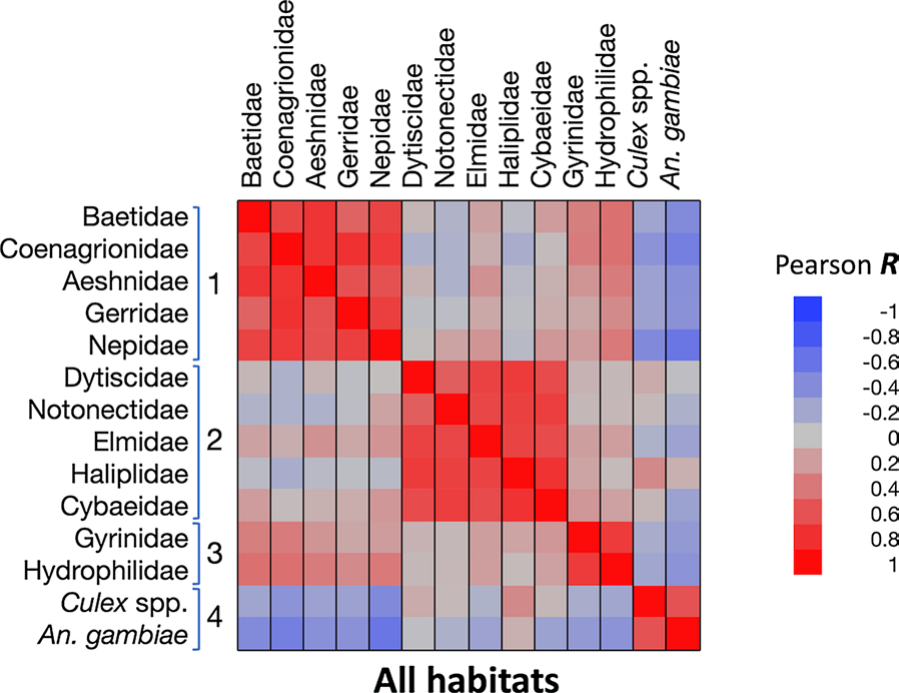
Correlation clustering analysis of the overall habitats based on Pearson pairwise correlation

Across all habitats there was a moderate positively correlated abundance of *An. gambiae* s.l. with *Culex* spp., thus highlighting that both taxa are rare in streams but plentiful in ponds, roadside ditches and temporary pools (Additional file 2: Table S3). There was also a weak but significant positive correlation with Dytiscidae, which is often present with *An. gambiae* s.l. There was significant abundance of *An. gambiae* s.l. negatively correlated with all other aquatic predatory taxa that are typically abundant in ponds and streams but not temporary habitats, except Cybaeidae. Clustering correlation analyses across habitats explained 77.3% of the variation in the data. Four clusters were identified, with a mean proportion of cluster variation explaining 79.1% (± 5.4 SD) and good overall fit illustrated by a low average 1 − *R*^2^ ratio [(1 − *R*^2^ with own cluster)/(1 − *R*^2^ with next closest)] equal to 0.261 (± 0.069 SD) (Fig. 7, Additional file 2: Table S4). Overall, this analysis mirrored the pairwise correlations between taxa and the two mosquito taxa clustered together due to broad similarities in habitat type preference and partial niche overlap.

**Fig. 8:**
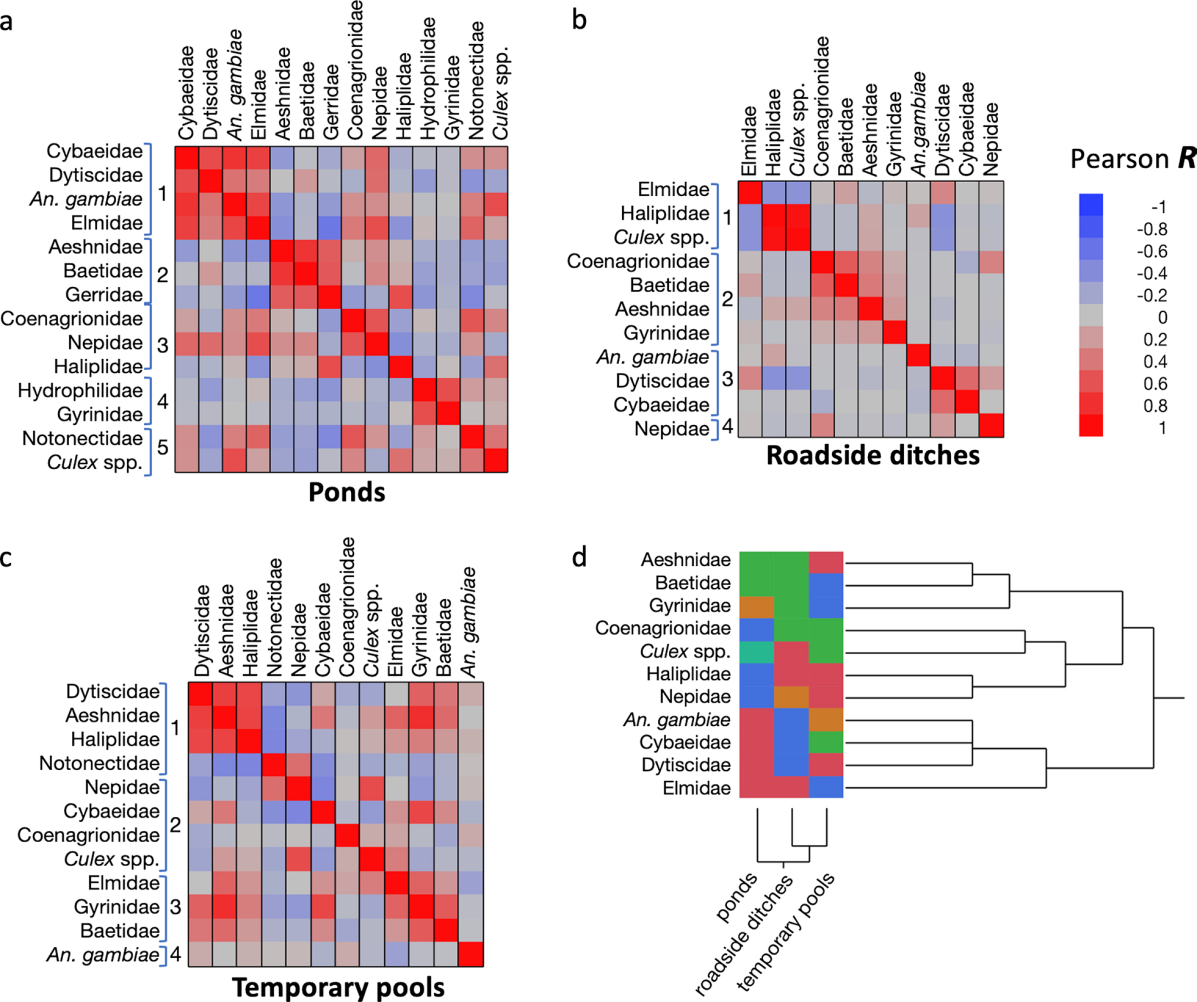
Correlation clustering analyses for ponds (**a**), roadside ditches (**b**) temporary pools (**c**) and the three habitat types combined (**d**)

Next, pairwise correlations between taxa and cluster correlation analyses were performed using the microhabitat (quadrat) data for each habitat type, except for streams, where *An. gambiae* s.l. was very rarely found. In pond microhabitats, there was a moderate positively correlated abundance of *An. gambiae* s.l. with Dytiscidae, Coenagrionidae, Cybaeidae, Culicidae, Elmidae and Nepidae. It also correlated negatively with Gerridae (water striders), Haliplidae and Baetidae. The clustering of all pairwise correlations resulted in five clusters and 71.3% of the variance explained, an average of 72.0% (± 7.4 SD) cluster variance explained, and a mean 0.353 (± 0.157 SD) 1 − *R*^2^ ratio (Fig. 8a, Additional file 2: Table S3). *Anopheles gambiae* s.l. clustered with Dytiscidae, Cybaeidae and Elmidae. *Culex* spp. was assigned to a different cluster with Notonectidae (Fig. 8a, Additional file 2: Table S3).

In roadside ditches, *An. gambiae* s.l. abundance correlated significantly with Dytiscidae, and there was a weak significant negative correlation with Baetidae (Additional file 2: Table S2). The clustering of correlations was not as pronounced, and accounted for only 60% of the variation explained and four clusters (cluster variance 67.9% ± 24 SD and 0.412 ± 0.307 SD). In that habitat, *An. gambiae* s.l. was grouped again with Dytiscidae and Cybaeidae, while *Culex* spp. were grouped with Elmidae and Haliplidae (Fig. 8b, Additional file 2: Table S3).

In temporary pools, clustering explained 62.3% of variation, four clusters (cluster variance 69.5% ± 21.7 SD and 0.453 ± 0.262 SD). In temporary pools, *An. gambiae* s.l.'s microhabitat use did not cluster with any of the other taxa. Its abundance did not correlate significantly with other taxa except for a weak negative correlation with Baetidae (Fig. 8c, Additional file 2: Tables S3 and S4). Finally, a two-way higher-level hierarchical clustering analysis based on the cluster membership generated by the three cluster correlation analyses for each habitat type confirmed that the two taxa whose use of aquatic microhabitats most closely matched that of *An. gambiae* s.l. were Cybaeidae and Dytiscidae (Fig. 8d).

For these two taxa and *Culex* spp., the level of association between their presence and absence across all quadrats from the two habitats preferred by *An. gambiae* s.l. (roadside ditches and temporary pools) was also tested. Overall, Dytiscidae co-occurred with *An. gambiae* s.l. in 73.1% of quadrats, and their presence/absence were significantly associated (Chi-square likelihood ratio test: *χ*^2^ = 24.6, *P* < 0.615). In contrast, Cybaeidae co-occurred with *An. gambiae* s.l. only in 5.7% of microhabitats, and their presence/absence was not significantly negatively associated (Chi-square likelihood ratio: *χ*^2^ = 0.56, *P* = 0.456). It is noteworthy that while the cluster correlation analyses did not reveal a strong similarity in correlation profiles of *An. gambiae* s.l. and *Culex* spp., the two taxa co-occurred in 67.1% of the quadrats sampled, and they were more likely to occur together than by chance (Chi-square likelihood ratio: *χ*^2^ = 23.1, *P* < 0.001).

## Discussion

Kibuye and Kayonjo are among the most malaria-prone villages in central Uganda due to the high abundance of *An. gambiae* s.l. [37, 47]. These villages are areas of intense malaria transmission, hence the focus for an investigation on the ecology of *An. gambiae* s.l. breeding habitats. The results provide the first account of the diversity and abundance of potential predators (aquatic insects that prey upon *An. gambiae* s.l. larvae) and competitors (other mosquito genera which share the same food item) of *An. gambiae* s.l. larvae in the different aquatic habitats in Uganda.

In this study, roadside ditches had the highest mean temperature, pH, EC and TDS, followed by intermediate values for temporary pools, with ponds and streams having the lowest parameter values. The prevailing water physico-chemical parameters of aquatic habitats influenced the insect taxa's distribution and abundance, including *An. gambiae* s.l. [48]. This study clearly shows that *An. gambiae* s.l. preferred temporary pools followed by roadside ditches, two habitats frequently shared with Culicinae mosquito larvae. These habitats were sometimes also used by various arthropod macroinvertebrates such as Dytiscidae, Nepidae, Notonectidae, Haliplidae, Elmidae, Baetidae and Cybaeidae. Previous studies have also reported that anopheline larvae often coexist with other macroinvertebrate predators [27, 49]. It has been suggested that *An. gambiae* s.l. larvae employ adaptive anti-predatory diving behaviour when sharing breeding habitats with aquatic predators [50]. Ponds had very few *An. gambiae* s.l. larvae, but very high numbers of Dytiscidae and Notonectidae. On the other hand, streams contained almost no *An. gambiae* s.l. larvae, but very high numbers of Baetidae, Coenagrionidae and Aeshnidae. In this study, we used a random sampling approach which, unavoidably, led to some quadrats being harder to sample with the standard dipper. In addition, habitats such as ponds and streams often had emergent vegetation that could have been used as a refuge by some macroinvertebrate taxa during sampling. This could have caused some bias in the habitat taxa profile reported in this study. However, we were highly confident that the large number of quadrats sampled per habitat ensured that major differences in taxa abundance and diversity were adequately detected.

The fact that *An. gambiae* s.l. larvae were common in temporary habitats and absent from permanent ones suggests that *An. gambiae* s.l. might have evolved to use temporal larval habitats to avoid predation and competition. Indeed, susceptibility to predation is one of the main forces structuring species communities among aquatic habitats [51]. The study has also shown that temporary pool breeding habitats and roadside ditches were the warmest and are therefore particularly suited for short multiple generation cycles such as that of mosquitoes.

Variation in the abundance and diversity of some aquatic insect taxa across habitat types and months was also largely associated with water persistence in each habitat type. High numbers of Gyrinidae and Hydrophilidae were found in the ponds during the start and peak of the short rainy season in October and November. High numbers of Baetidae were found at the end of the rainy season in December. This was probably due to water persisting in this habitat well beyond the entire sampling period and possibly for the better part of the year. The permanence of ponds can explain the highest diversity of insect taxa found in this habitat compared to streams, temporary pools and roadside ditches, a pattern that has been highlighted in other studies [21, 52–54].

The increase in numbers of Aeshnidae, Baetidae, Coenagrionidae and Nepidae in roadside ditches from the start to the end of the rainy season suggest that, even in such ephemeral habitats, water was present long enough for some taxa to possibly sustain a new generation and increase in numbers [55]. The nymphs of some Baetidae can complete their life cycle and emerge within a few weeks to contribute to the next generation, which will lay new eggs into the rain-filled breeding habitats. Other taxa, such as Aeshnidae, Coenagrionidae and Nepidae, have slower development, and their larvae must survive through the repeated wet and dry cycles of semi-permanent and temporal aquatic habitats [56, 57]. For these species, the seasonal increase in numbers within the short rainy season suggests that semi-permanent and temporal aquatic habitats may be visited by adults who emerged from more permanent aquatic habitats or survived the dry season, waiting for the next rainy season to reproduce. For such species, laying eggs in more ephemeral habitats may be part of an oviposition bet-hedging strategy that creates opportunities for some offspring to develop in environments with lower-level competition and predation from other aquatic predators than in ponds.

Interestingly, we found a few larvae of *An. gambiae* s.l. in streams in November, following an interruption in rainfall, which resulted in the creation of sun-exposed puddle-like habitats suitable for *An. gambiae* s.l. [58]. In those puddle-like habitats, *An. gambiae* s.l. larvae were often found with Hydrophilidae, which mainly breed in permanent water bodies [59]. The *An. gambiae* s.l. larvae may have been trapped in these temporarily created breeding habitats. Such a rain pattern could also explain the appearance of Cybaeidae, Gyrinidae and Haliplidae towards the end of the short rainy season in streams.

Across all habitats, the abundance of *An. gambiae* s.l. gave a significantly positive correlation with the *Culex* spp. This can largely be explained by the fact that both mosquito taxa prefer roadside ditches and temporary pools over ponds and streams. Despite this apparent niche overlap, there were differences between the two taxa. Notably, *Culex* spp. was regularly found in streams, where *An. gambiae* s.l. is extremely rare. It was also more common in ponds and less frequent in roadside ditches compared to *An. gambiae* s.l. Thus, there is only partial niche overlap between these taxa in terms of habitat preferences.

This study pushed the analyses further by clustering all pairwise correlations among aquatic invertebrates to identify those whose patterns of abundance in samples from different microhabitats/quadrats most closely matched. This approach, applied to all quadrats from all habitats, generally grouped taxa whose abundance and breeding site preferences correlated broadly across all habitats. In this instance, *An. gambiae* s.l. and *Culex* spp*.* clustered together. However, the congruence between both analytical approaches stopped when conducted within habitat type, the spatial scale most likely to highlight predators and competitors that might interact with *An. gambiae* s.l. In ponds, *An. gambiae* s.l. abundance showed a moderate positive correlation with Dytiscidae, Cybaeidae, Coenagrionidae, Nepidae, Elmidae and *Culex* spp., but a significant negative correlation with other taxa except Hydrophilidae and Aeshnidae. The cluster correlation analyses grouped *An. gambiae* s.l. with Dytiscidae, Cybaeidae and Elmidae, suggesting that these tend to be captured within the same microhabitats within ponds, and in this particular instance, the herbaceous margins of ponds. *Culex* spp*.* were grouped into a separate cluster with Notonectidae, a taxon that prefers deeper water further from the ponds' edges. Overall, the cluster correlation analyses underlined the pond's heterogeneity, with different microhabitats populated by different assemblages of aquatic invertebrate taxa. The same analyses conducted on the data from roadside ditches and temporary rain pools again showed that the microhabitat usage by *An. gambiae* s.l. was most similar to Dytiscidae and Cybaeidae. *Culex* spp. was less frequent than *An. gambiae* s.l. in such habitats, and it did not cluster with *An. gambiae* s.l., suggesting again that it uses different areas of the aquatic habitats. *Culex* spp*.* are known to prefer habitats richer in organic matter, and *An. gambiae* s.l. cleaner water. Ecological niche partitioning may thus explain why, despite their similar life cycle, the two mosquito taxa did not cluster together [60]. It should be noted that, whilst *Culex quinquefasciatus* is usually the dominant species in domestic and peridomestic settings [61], we cannot dismiss the possibility that some of the sylvatic *Culex* spp. found in Uganda were also present in our samples [62, 63].

Thus, the overall patterns of temporal and spatial dynamics of *An. gambiae* s.l. larvae and other aquatic invertebrates suggest that Dytiscidae and, in some microhabitats, Cybaeidae could be important predators of *An. gambiae* s.l. This is based on the assumption that if two taxa compete or interact as prey and predator, they are expected to be found together in breeding habitats [64–66]. Moreover, it further assumes that, as in other predator–prey systems, aquatic invertebrate predators do not wipe out their prey completely, resulting in predator and prey abundance being positively correlated [67]. Nevertheless, because the data are based on observations and correlational analyses, further studies are needed to test the strength of these interactions experimentally. Also, although we were able to generate a substantial amount of information from the data collected during the short rainy season of October–December, the dynamics taking place during the rest of the year and particularly during the other rainy season of March–May remains to be unraveled. This was the limitation of this study.

In our samples, 80% of the Dytiscidae belonged to the genus *Cybister,* and the remaining samples were from the genus *Rhantus*. Over 70% of individuals had reached adult stages, and 30% were larvae. The potential use of Dytiscidae for mosquito control has been examined in experiments focusing on two Asian Dytiscidae species [68, 69]. These studies demonstrated that water beetles are very effective predators and can consume dozens of mosquito larvae per day. Additionally, adult Dytiscidae are good fliers and can easily find new habitats. Furthermore, their larvae have a fast developmental rate, can undergo metamorphosis and remain dormant in the mud of dried-up temporary larval habitats till the next rains. These traits make water beetles particularly adapted to the preferred habitats of *An. gambiae* s.l. The Cybaeidae are much less frequent than Dytiscidae but share the shallow herbaceous margins of ponds and temporary pools with *An. gambiae* s.l. larvae. Cluster correlation analyses highlighted this similarity of microhabitat usage, giving confidence in the sampling and analytical approach of the study. In retrospect, it would be recommended that when a similar sampling approach is used, microhabitats/quadrats could be further characterized in terms of water depth, distance from the water edge and aquatic vegetation, which are likely important predictors of the aquatic arthropod communities. While unable to fly, Cybaeidae are thought to reach new habitats via the dispersal of juveniles which are thought to use silk threads to catch the wind [70, 71]. Given the narrower set of conditions in which this taxon was found in numbers alongside *An. gambiae* s.l., and taking into account their complex life cycle, it has less potential for biocontrol developments.

## Conclusions

Mosquito breeding habitats such as streams, ponds, roadside ditches and temporary pools differed in water physico-chemical parameters (temperature, pH, TDS and EC). They differed in the abundance and diversity of macroinvertebrate predators and competitor insect taxa and how these are associated with *An. gambiae* s.l. larvae. Ponds and streams were the most preferred breeding habitats for most predators and competitors, while temporary pools and roadside ditches were preferred by *An. gambiae* s.l. larvae. In these habitats, there were partial niche overlaps between *An. gambiae* s.l. larvae and *Culex* spp., but there was no evidence of competition between the two taxa. Overall, the predators that most closely shared *An. gambiae* s.l.'s temporal and spatial dynamics across and within *An. gambiae* s.l. larval habitats were Dytiscidae and, in some microhabitats, Cybaeidae. The strength of these prey–predator relationships and their potential for mosquito larvae's biocontrol in the African setting remains to be demonstrated through controlled experimental studies. Also, we recommend further studies using a similar method for over 1–2 years. This would allow for a reflection of information variations across seasons.

## Supplementary Information

**Additional file1**:** Figure S1**. Neighbour-joining cluster analyses based on (**a**) physico-chemical parameters and (**b**) aquatic insect taxa abundance of two quadrats from each of the 32 surveyed habitats. Samples from the two quadrats were collected one month apart (October–November). The tree based on chemical parameters had much shallower distal branches, and several pairs of quadrats from the same breeding habitat were nearest neighbours (highlighted). In comparison, the tree based on species abundance had deeper distal branching, and none of the quadrats sampled from the same habitat was nearest neighbours.

**Additional file 2**:** Table S1**. Mean and median abundance, diversity and percentage frequency of aquatic insect taxa across habitat types in Kibuye.** Table S2**. Mean and median abundance, diversity and percentage frequency of aquatic insect taxa across habitat types in Kayonjo. **Table S3**. Pairwise correlations between the abundance of *Anopheles gambiae* sensu lato larvae and the abundance of aquatic predators and competitors across all habitats and for each habitat type.** Table S4**. Cluster membership based on heat map analyses for the correlations across all habitats and for each habitat type.

## Data Availability

The data is available upon request to the corresponding authors.

## Abbreviations

s.l.: Sensu lato
TDS: Total dissolved solids
EC: Electrical conductivity
s.s.: Sensu stricto
pH: Potential hydrogen
LVB: Lake Victoria Basin
PCA: Principal component analysis
