# Supplementary figures and images for: Predatory and competitive interaction in Anopheles gambiae sensu lato larval breeding habitats in selected villages of central Uganda
Source: Parasit Vectors. 2021 Aug 21;14:420. doi: 10.1186/s13071-021-04926-9 (PMC8380324; doi:10.1186/s13071-021-04926-9)

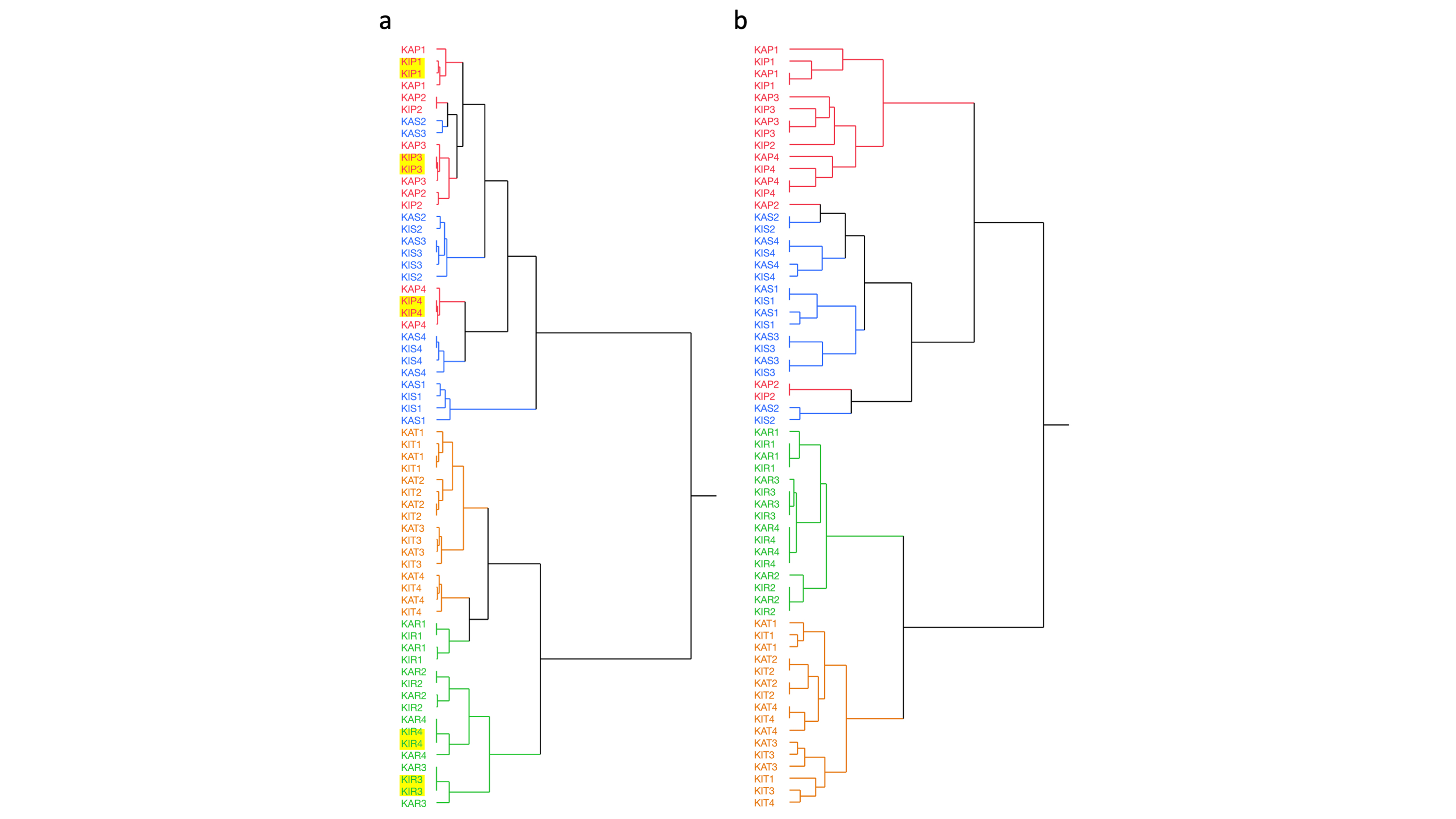

Supplement: Supplementary file 1 — Additional file1: Figure S1. Neighbour-joining cluster analyses based on (a) physico-chemical parameters and (b) aquatic insect taxa abundance of two quadrats from each of the 32 surveyed habitats. Samples from the two quadrats were collected one month apart (October–November). The tree based on chemical parameters had much shallower distal branches, and several pairs of quadrats from the same breeding habitat were nearest neighbours (highlighted). In comparison, the tree based on species abundance had deeper distal branching, and none of the quadrats sampled from the same habitat was nearest neighbours. [file 13071_2021_4926_MOESM1_ESM.tif]
