# Supplementary material for: Predatory and competitive interaction in Anopheles gambiae sensu lato larval breeding habitats in selected villages of central Uganda
Source: Parasit Vectors. 2021 Aug 21;14:420. doi: 10.1186/s13071-021-04926-9 (PMC8380324; doi:10.1186/s13071-021-04926-9)
Supplement: Supplementary file 2 — Additional file 2: Table S1. Mean and median abundance, diversity and percentage frequency of aquatic insect taxa across habitat types in Kibuye. Table S2. Mean and median abundance, diversity and percentage frequency of aquatic insect taxa across habitat types in Kayonjo. Table S3. Pairwise correlations between the abundance of Anopheles gambiae sensu lato larvae and the abundance of aquatic predators and competitors across all habitats and for each habitat type. Table S4. Cluster membership based on heat map analyses for the correlations across all habitats and for each habitat type. [file 13071_2021_4926_MOESM2_ESM.docx]

**Additional file 2: Table S1.** Mean and Median abundance, diversity and percentage frequency of aquatic insect taxa across habitat types in Kibuye

| Taxa (common name) | Kibuye | | | | | | | | | | | |
| --- | --- | --- | --- | --- | --- | --- | --- | --- | --- | --- | --- | --- |
|  | Ponds | | | Roadside ditches | | | Temporary pools | | | Streams | | |
|  | Mean | Median | Freq (%) | Mean | Median | Freq (%) | Mean | Median | Freq (%) | Mean | Median | Freq (%) |
| *An. gambiae* s.l. (mosquito) | 0.5 | 0 (0-2) | 37.5 | 1 | 1 (0-2) | 75.0 | 2.84 | 3 (0-8) | 99.1 | 0.03 | 0 (0-2) | 0.2 |
| Aeshnidae (dragonfly) | 0.93 | 1 (0-8) | 65.6 | 0.02 | 0 (0-1) | 2.1 | 0.25 | 0 (0-1) | 23.1 | 2.88 | 2 (1-8) | 100 |
| Baetidae (mayflies) | 1.52 | 1 (0-8) | 66.7 | 0.04 | 0 (0-1) | 4.2 | 0.5 | 0.5 (0-1) | 50.0 | 5.88 | 5.5 (3-8) | 100 |
| Coenagrionidae (damselflies) | 1.05 | 1 (0-5) | 64.6 | 0.03 | 0 (0-1) | 3.1 | 0.38 | 0 (0-1) | 37.5 | 3 | 3 (1-5) | 100 |
| *Culex* spp. (mosquito) | 1.63 | 1.5 (0-3) | 75.0 | 0.88 | 1 (0-2) | 62.5 | 3.52 | 3 (1-8) | 100 | 0.5 | 0 (0-3) | 36.5 |
| Cybaeidae (water spider) | 1 | 1 (0-2) | 75.0 | 0 | 0 (0-0) | 0.0 | 0.13 | 0 (0-1) | 12.5 | 0 | 0 (0-0) | 0.0 |
| Dytiscidae (predaceous water beetles) | 7.7 | 7.5 (5-13) | 100 | 1.13 | 1 (0-3) | 62.5 | 3.63 | 4 (1-6) | 100 | 1.5 | 1.5 (0-4) | 62.5 |
| Elmidae (riffle beetles) | 2.5 | 2.5 (1-4) | 100 | 0.13 | 0 (0-1) | 12.5 | 0.38 | 0 (0-2) | 25.0 | 0.75 | 1 (0-2) | 62.5 |
| Gerridae (pond skaters) | 0.7 | 1 (0-2) | 52.1 | 0 | 0 (0-0) | 0.0 | 0 | 0 (0-0) | 0.0 | 1.5 | 1.5 (1-2) | 100 |
| Gyrinidae (whirligig beetles) | 0.88 | 0.5 (0-5) | 50.0 | 0.08 | 0 (0-1) | 8.3 | 0.05 | 0 (0-1) | 5.2 | 1.16 | 1 (0-5) | 59.4 |
| Haliplidae (crawling water beetles) | 2.63 | 2.5 (1-5) | 100 | 0.13 | 0 (0-1) | 12.5 | 0.63 | 0.5 (0-2) | 50.0 | 0 | 0 (0-0) | 0 |
| Hydrophilidae (water scavenger beetles) | 0.38 | 0 (0-2) | 33.3 | 0 | 0 (0-0) | 0 | 0 | 0 (0-0) | 0 | 0.66 | 1 (0-2) | 58.3 |
| Nepidae (water scorpions) | 0.9 | 1 (0-4) | 65.6 | 0.02 | 0 (0-1) | 1.0 | 0.25 | 0 (0-1) | 25.0 | 2.5 | 2.5 (1-4) | 100 |
| Notonectidae (backswimmers) | 4.38 | 5 (2-6) | 100 | 0 | 0 (0-0) | 0 | 0.38 | 0 (0-2) | 25.0 | 0.88 | 1 (0-2) | 62.5 |
| Taxa present | 14 | |  | 10 | |  | 12 | |  | 12 | |  |
| Shannon index of diversity | 1.11 (1.10-1.12) | |  | 0.69 (0.59-0.71) | |  | 0.93 (0.92-0.94) | |  | 1.11 (1.11-1.13) | |  |
|  |  |  |  |  |  |  |  |  |  |  |  |  |

Median abundances are presented for each taxon with minimum and maximum values in brackets. For diversity index, the median is shown with 27-75 quartiles in bracket. Frequency is equivalent to proportion of quadrats from which the taxa were collected.

**Additional file 2: Table S2.** Mean and median abundance, diversity and percentage frequency of aquatic insect taxa across habitat types in Kayonjo

| Taxa (common name) | Kayonjo | | | | | | | | | | | |
| --- | --- | --- | --- | --- | --- | --- | --- | --- | --- | --- | --- | --- |
|  | Ponds | | | Roadside ditches | | | Temporary pools | | | Streams | | |
|  | Mean | Median | Freq (%) | Mean | Median | Freq (%) | Mean | Median | Freq (%) | Mean | Median | Freq (%) |
| *An. gambiae* s.l. (mosquito) | 0.5 | 0 (0-2) | 37.5 | 1 | 1 (0-2) | 75.0 | 2.6 | 2 (0-8) | 99.1 | 0.02 | 0 (0-2) | 1.0 |
| Aeshnidae (dragonfly) | 0.93 | 1 (0-8) | 66.3 | 0.02 | 0 (0-1) | 2.1 | 0.25 | 0 (0-1) | 25.0 | 2.88 | 2 (1-8) | 100 |
| Baetidae (mayflies) | 1.52 | 1 (0-8) | 65.6 | 0.04 | 0 (0-1) | 4.2 | 0.5 | 0.5 (0-1) | 50.0 | 5.88 | 5.5 (3-8) | 100 |
| Coenagrionidae (damselflies) | 1.05 | 1 (0-5) | 65.6 | 0.03 | 0 (0-1) | 3.1 | 0.38 | 0 (0-1) | 37.5 | 3 | 3 (1-5) | 100 |
| *Culex* spp. (mosquito) | 1.63 | 1.5 (0-3) | 75.0 | 0.88 | 1 (0-2) | 62.5 | 3.47 | 3 (0-8) | 98.1 | 0.52 | 0 (0-4) | 36.5 |
| Cybaeidae (water spider) | 0.92 | 1 (0-2) | 68.8 | 0.01 | 0 (0-1) | 1.04 | 0.11 | 0 (0-1) | 11.5 | 0.08 | 0 (0-2) | 6.3 |
| Dytiscidae (predaceous water beetles) | 7.38 | 7 (1-12) | 100 | 1.05 | 1 (0-3) | 59.4 | 3.53 | 4 (1-6) | 100 | 1.41 | 1 (0-4) | 59.4 |
| Elmidae (riffle beetles) | 2.35 | 2 (0-4) | 96.9 | 0.15 | 0 (0-3) | 13.5 | 0.35 | 0 (0-2) | 23.9 | 0.9 | 1 (0-4) | 65.6 |
| Gerridae (pond skaters) | 0.7 | 1 (0-2) | 52.1 | 0 | 0 (0-0) | 0 | 0 | 0 (0-0) | 0 | 1.5 | 1.5 (1-2) | 100 |
| Gyrinidae (whirligig beetles) | 0.78 | 0 (0-5) | 46.9 | 0.09 | 0 (0-1) | 9.4 | 0.05 | 0 (0-1) | 5.2 | 1.16 | 1 (0-5) | 59.4 |
| Haliplidae (crawling water beetles) | 2.41 | 2 (0-5) | 91.7 | 0.17 | 0 (0-2) | 15.6 | 0.58 | 0 (0-2) | 46.9 | 0.22 | 0 (0-5) | 8.3 |
| Hydrophilidae (water scavenger beetles) | 0.28 | 0 (0-2) | 25.0 | 0 | 0 (0-0) | 0 | 0 | 0 (0-0) | 0 | 0.66 | 1 (0-2) | 58.3 |
| Nepidae (water scorpions) | 0.9 | 1 (0-0.4) | 65.6 | 0.02 | 0 (0-1) | 1.0 | 0.25 | 0 (0-1) | 25.0 | 2.5 | 2.5 (1-4) | 100 |
| Notonectidae (backswimmers) | 4.06 | 5 (0-6) | 97.9 | 0 | 0 (0-0) | 0 | 0.49 | 0 (0-4) | 29.2 | 0.84 | 1 (0-3) | 59.4 |
| Taxa present | 14 | |  | 11 | |  | 12 | |  | 14 | |  |
| Shannon index of diversity | 1.09 (1.09-1.11) | |  | 0.73 (0.69-0.87) | |  | 1.02 (1.01-1.08) | |  | 1.11 (1.03-1.14) | |  |

Median abundances are presented for each taxon with minimum and maximum values in brackets. For diversity index, the median is shown with 27-75 quartiles in bracket. Frequency is equivalent to proportion of quadrats from which the taxa were collected.

Additional file 2: Table S3. Pairwise correlations between the abundance of *Anopheles gambiae* sensu lato larvae and the abundance of aquatic predators and competitors across all habitats and for each habitat type

| Habitats | | Mosquito larvae | Aquatic insect taxa | | Pearson correlation coefficient | *P*-value |
| --- | --- | --- | --- | --- | --- | --- |
| All habitats | | *An. gambiae* s.l. | Nepidae | | -0.41 | <0.001 |
|  | | *An. gambiae* s.l. | Baetidae | | -0.501 | <0.001 |
|  | | *An. gambiae* s.l. | Coenagrionidae | -0.374 | | <0.001 |
|  | | *An. gambiae* s.l. | Aeshnidae | | -0.363 | <0.001 |
|  | | *An. gambiae* s.l. | Gerridae | | -0.573 | <0.001 |
|  | | *An. gambiae* s.l. | *Culex* spp. | | 0.6 | <0.001 |
|  | | *An. gambiae* s.l. | Cybaeidae | | -0.023 | 0.531 |
|  | | *An. gambiae* s.l. | Haliplidae | | -0.131 | 0.001 |
|  | | *An. gambiae* s.l. | Elmidae | | -0.242 | <0.001 |
|  | | *An. gambiae* s.l. | Dytiscidae | | 0.101 | 0.005 |
|  | | *An. gambiae* s.l. | Notonectidae | | -0.244 | <0.001 |
|  | | *An. gambiae* s.l. | Gyrinidae | | -0.295 | <0.001 |
|  | | *An. gambiae* s.l. | Hydrophilidae | | -0.347 | <0.001 |
| Ponds | | *An. gambiae* s.l. | Nepidae | | 0.322 | <0.001 |
|  | | *An. gambiae* s.l. | Baetidae | | -0.364 | <0.001 |
|  | | *An. gambiae* s.l. | Coenagrionidae | | 0.539 | <0.001 |
|  | | *An. gambiae* s.l. | Aeshnidae | | -0.111 | 0.13 |
|  | | *An. gambiae* s.l. | Gerridae | | -0.534 | <0.001 |
|  | | *An. gambiae* s.l. | *Culex* spp. | | 0.414 | <0.001 |
|  | | *An. gambiae* s.l. | Cybaeidae | | 0.593 | <0.001 |
|  | | *An. gambiae* s.l. | Haliplidae | | -0.377 | <0.001 |
|  | | *An. gambiae* s.l. | Elmidae | | 0.355 | <0.001 |
|  | | *An. gambiae* s.l. | Dytiscidae | | 0.665 | <0.001 |
|  | | *An. gambiae* s.l. | Notonectidae | | 0.104 | 0.152 |
|  | | *An. gambiae* s.l. | Gyrinidae | | 0.033 | 0.647 |
|  | | *An. gambiae* s.l. | Hydrophilidae | | -0.075 | 0.303 |
| Roadside ditches | | *An. gambiae* s.l. | Nepidae | | 0 | 1 |
|  | | *An. gambiae* s.l. | Baetidae | | -0.147 | 0.041 |
|  | | *An. gambiae* s.l. | Coenagrionidae | | 0 | 1 |
|  | | *An. gambiae* s.l. | Aeshnidae | | 0 | 1 |
|  | | *An. gambiae* s.l. | *Culex* spp. | | 0 | 1 |
|  | | *An. gambiae* s.l. | Cybaeidae | | -0.102 | 0.158 |
|  | | *An. gambiae* s.l. | Haliplidae | | -0.04 | 0.581 |
|  | | *An. gambiae* s.l. | Elmidae | | -0.021 | 0.776 |
|  | | *An. gambiae* s.l. | Dytiscidae | | 0.477 | <0.001 |
| Temporary pools | *An. gambiae* s.l. | Nepidae | | 0.043 | 0.556 |  |
|  | *An. gambiae* s.l. | Baetidae | | -0.248 | 0.001 |  |
|  | *An. gambiae* s.l. | Coenagrionidae | | -0.118 | 0.103 |  |
|  | *An. gambiae* s.l. | Aeshnidae | | 0.123 | 0.089 |  |
|  | *An. gambiae* s.l. | *Culex* spp. | | 0.125 | 0.085 |  |
|  | *An. gambiae* s.l. | Cybaeidae | | -0.089 | 0.218 |  |
|  | *An. gambiae* s.l. | Haliplidae | | 0.006 | 0.931 |  |
|  | *An. gambiae* s.l. | Elmidae | | -0.049 | 0.502 |  |
|  | *An. gambiae* s.l. | Dytiscidae | | 0.089 | 0.219 |  |
|  | *An. gambiae* s.l. | Notonectidae | | 0.022 | 0.764 |  |
|  | *An. gambiae* s.l. | Gyrinidae | | -0.024 | 0.738 |  |

**Additional file 2: Table S4.** Cluster membership based on heatmap analyses for the correlations across all habitats and for each habitat type

| Habitats | Cluster | Members | *R*^2^ own cluster | *R*^2^ next closest | 1- *R*^2^ ratio |
| --- | --- | --- | --- | --- | --- |
| All habitats | 1 | Baetidae | 0.850 | 0.233 | 0.196 |
|  | 1 | Coenagrionidae | 0.761 | 0.147 | 0.281 |
|  | 1 | Gerridae | 0.751 | 0.274 | 0.343 |
|  | 1 | Aeshnidae | 0.699 | 0.129 | 0.346 |
|  | 1 | Nepidae | 0.706 | 0.189 | 0.363 |
|  | 2 | Notonectidae | 0.762 | 0.047 | 0.250 |
|  | 2 | Elmidae | 0.737 | 0.044 | 0.275 |
|  | 2 | Haliplidae | 0.693 | 0.009 | 0.310 |
|  | 2 | Cybaeidae | 0.680 | 0.014 | 0.325 |
|  | 2 | Dytiscidae | 0.673 | 0.086 | 0.358 |
|  | 3 | Gyrinidae | 0.863 | 0.094 | 0.151 |
|  | 3 | Hydrophilidae | 0.863 | 0.206 | 0.173 |
|  | 4 | *Culex* spp. | 0.725 | 0.115 | 0.311 |
|  | 4 | *An. gambiae* s.l. | 0.690 | 0.263 | 0.421 |
|  | 4 | Sphaerolichidae | 0.490 | 0.098 | 0.565 |
| Ponds | 1 | *An. gambiae* s.l. | 0.733 | 0.231 | 0.347 |
|  | 1 | Cybaeidae | 0.681 | 0.140 | 0.370 |
|  | 1 | Dytiscidae | 0.490 | 0.039 | 0.530 |
|  | 1 | Elmidae | 0.505 | 0.117 | 0.561 |
|  | 2 | Aeshnidae | 0.826 | 0.083 | 0.190 |
|  | 2 | Baetidae | 0.808 | 0.082 | 0.210 |
|  | 2 | Gerridae | 0.634 | 0.197 | 0.456 |
|  | 3 | Coenagrionidae | 0.800 | 0.264 | 0.272 |
|  | 3 | Nepidae | 0.800 | 0.293 | 0.283 |
|  | 4 | Hydrophilidae | 0.814 | 0.023 | 0.191 |
|  | 4 | Gyrinidae | 0.814 | 0.083 | 0.203 |
|  | 5 | *Culex* spp. | 0.713 | 0.218 | 0.367 |
|  | 5 | Notonectidae | 0.713 | 0.424 | 0.499 |
|  | 6 | Sphaerolichidae | 0.673 | 0.088 | 0.359 |
|  | 6 | Haliplidae | 0.673 | 0.112 | 0.368 |
| Roadside ditches | 1 | Elmidae | 0.919 | 0.041 | 0.084 |
|  | 1 | Haliplidae | 0.921 | 0.069 | 0.085 |
|  | 1 | *Culex* spp. | 0.332 | 0.179 | 0.814 |
|  | 2 | Coenagrionidae | 0.678 | 0.012 | 0.326 |
|  | 2 | Baetidae | 0.618 | 0.112 | 0.430 |
|  | 2 | Aeshnidae | 0.499 | 0.021 | 0.512 |
|  | 2 | Gyrinidae | 0.152 | 0.014 | 0.860 |
|  | 3 | *An. gambiae* s.l. | 0.718 | 0.004 | 0.283 |
|  | 3 | Dytiscidae | 0.704 | 0.163 | 0.354 |
|  | 3 | Cybaeidae | 0.087 | 0.003 | 0.916 |
|  | 4 | Nepidae | 1.000 | 0.012 | 0.000 |
|  | 5 | Sphaerolichidae | 1.000 | 0.045 | 0.000 |
| Temporary pools | 1 | Elmidae | 0.863 | 0.381 | 0.221 |
|  | 1 | Coenagrionidae | 0.574 | 0.164 | 0.509 |
|  | 1 | Gyrinidae | 0.468 | 0.154 | 0.628 |
|  | 1 | Baetidae | 0.406 | 0.106 | 0.665 |
|  | 2 | Cybaeidae | 0.819 | 0.034 | 0.187 |
|  | 2 | Nepidae | 0.819 | 0.099 | 0.200 |
|  | 3 | Dytiscidae | 0.781 | 0.053 | 0.231 |
|  | 3 | Aeshnidae | 0.702 | 0.142 | 0.348 |
|  | 3 | Haliplidae | 0.802 | 0.542 | 0.432 |
|  | 3 | Notonectidae | 0.366 | 0.094 | 0.700 |
|  | 4 | *An. gambiae* s.l. | 1.000 | 0.018 | 0.000 |
|  | 5 | *Culex* spp. | 0.521 | 0.016 | 0.486 |
|  | 5 | Sphaerolichidae | 0.521 | 0.045 | 0.501 |
